# Familial risk for major depression: differential white matter alterations in healthy and depressed participants

**DOI:** 10.1017/S003329172200188X

**Published:** 2023-08

**Authors:** Alexandra Winter, Katharina Thiel, Susanne Meinert, Hannah Lemke, Lena Waltemate, Fabian Breuer, Regina Culemann, Julia-Katharina Pfarr, Frederike Stein, Katharina Brosch, Tina Meller, Kai Gustav Ringwald, Florian Thomas-Odenthal, Andreas Jansen, Igor Nenadić, Axel Krug, Jonathan Repple, Nils Opel, Katharina Dohm, Elisabeth J. Leehr, Dominik Grotegerd, Harald Kugel, Tim Hahn, Tilo Kircher, Udo Dannlowski

**Affiliations:** 1 Institute for Translational Psychiatry, University of Münster, Münster, Germany; 2 Institute of Translational Neuroscience, University of Münster, Münster, Germany; 3 Department of Psychiatry und Psychotherapy, University of Marburg, Marburg, Germany; 4 Center for Mind, Brain and Behavior (CMBB), University of Marburg and Justus Liebig University Giessen, Marburg, Germany; 5 Department of Psychiatry and Psychotherapy, University of Bonn, Bonn, Germany; 6 University Clinic for Radiology, University of Muenster, Münster, Germany

**Keywords:** white matter, familial risk, major depressive disorder, diffusion tensor imaging

## Abstract

**Background:**

Major depressive disorder (MDD) has been associated with alterations in brain white matter (WM) microstructure. However, diffusion tensor imaging studies in biological relatives have presented contradicting results on WM alterations and their potential as biomarkers for vulnerability or resilience. To shed more light on associations between WM microstructure and resilience to familial risk, analyses including both healthy and depressed relatives of MDD patients are needed.

**Methods:**

In a 2 (MDD v. healthy controls, HC) × 2 (familial risk yes v. no) design, we investigated fractional anisotropy (FA) via tract-based spatial statistics in a large well-characterised adult sample (*N* = 528), with additional controls for childhood maltreatment, a potentially confounding proxy for environmental risk.

**Results:**

Analyses revealed a significant main effect of diagnosis on FA in the forceps minor and the left superior longitudinal fasciculus (*p*_tfce−FWE_ = 0.009). Furthermore, a significant interaction of diagnosis with familial risk emerged (*p*_tfce−FWE_ = 0.036) Post-hoc pairwise comparisons showed significantly higher FA, mainly in the forceps minor and right inferior fronto-occipital fasciculus, in HC with as compared to HC without familial risk (*p*_tfce−FWE_ < 0.001), whereas familial risk played no role in MDD patients (*p*_tfce−FWE_ = 0.797). Adding childhood maltreatment as a covariate, the interaction effect remained stable.

**Conclusions:**

We found widespread increased FA in HC with familial risk for MDD as compared to a HC low-risk sample. The significant effect of risk on FA was present only in HC, but not in the MDD sample. These alterations might reflect compensatory neural mechanisms in healthy adults at risk for MDD potentially associated with resilience.

## Introduction

Major depressive disorder (MDD) is a debilitating, life-quality diminishing, and often recurring mental disorder with a lifetime prevalence ranging from 10% to 29.9% (Kessler, Petukhova, Sampson, Zaslavsky, & Wittchen, 2012; for a meta-analysis, see Lim *et al*. 2018). Besides examining the course and symptomatology of the disorder itself, it is of high clinical relevance to identify risk factors contributing to the development of depressive psychopathology. Family history of MDD is a consistently replicated risk factor: Genetic approaches involving twin studies estimate a heritability of 30–40% (Kendler, Gatz, Gardner, & Pedersen, 2006; Sullivan, Michael Neale, & Kendler, 2000) and one of the largest genome-wide association studies has recently highlighted the complex genetic architecture of depression by identifying more than 200 genes associated with MDD (Howard *et al*. 2019). Moreover, parental depression increases the risk to develop MDD threefold, and was associated with a higher probability of recurring episodes, mortality and overall poorer functioning as compared to offspring of non-depressed parents (Hammen, Burge, Burney, & Adrian, 1990; Weissman *et al*. 2016a, 2016b). The period between 15 and 25 years of age is most critical to develop MDD for individuals with familial risk, but also without familial risk, as revealed in the 30-year follow-up study (Weissman *et al*. 2016b). Not only offspring, but first-degree relatives of MDD patients in general exhibit elevated rates of MDD as compared to individuals without a familial predisposition (Klein, Lewinsohn, Seeley, & Rohde, 2001).

Importantly, this familial risk entails environmental stressors, as genetic risk and in some cases, the emotionally and socially strenuous environment among depressed relatives, jointly contribute to psychopathology in offspring (Goodman & Gotlib, 1999) by, for instance, emotionally neglectful upbringing and other forms of childhood maltreatment (Lovejoy, Graczyk, O'Hare, & Neuman, 2000; Pawlby, Hay, Sharp, Waters, & Pariante, 2011).

Yet, not all healthy individuals exposed to depression in their familial surrounding manifest clinically relevant depressive symptoms, but are resilient to this risk. Resilience is understood as the dynamic ability to maintain well-being and mental health by activating protective resources, even when facing adversity, i.e., suffering, discomfort or a potentially traumatic event in life (Sisto *et al*. 2019; Windle, 2011). However, little is known regarding neurobiological differences between resilient individuals exposed to familial risk, and exposed individuals who developed MDD themselves. On the one hand, it could be speculated that familial risk exposure shapes brain structure and function towards alterations found in patient samples, suggesting risk-associated markers of vulnerability. On the other hand, the opposite might be the case, suggesting (over-)compensatory mechanisms related to resilience in exposed persons.

While some neuroimaging studies rather point towards a structural and functional neurobiological similarity of at-risk individuals and MDD patients, e.g., regarding reduced hippocampal volume (Amico *et al*. 2011; Peterson *et al*. 2009); and alterations in emotion-processing circuits (e.g. Gotlib, Joormann, & Foland-Ross, 2014; Opel *et al*. 2017), others have provided evidence for correlates of resilience by increased grey matter volume in the dorsolateral prefrontal cortex (Brosch *et al*. 2021). Despite the soaring awareness that differences in white matter (WM) fibre structure seem to be play a role in the pathophysiology of MDD (Murphy & Frodl, 2011; van Velzen *et al*. 2020), examining WM microstructure alterations in participants with familial risk has been neglected. The superior longitudinal fasciculus (SLF) is a major WM tract connecting the frontal lobe with posterior regions (Makris *et al*. 2005). While its function has not been fully established yet, it has been associated with depression (Murphy & Frodl, 2011), emotion processing (Koshiyama *et al*. 2020), and working memory (Karlsgodt *et al*. 2008). Other tracts which have been associated with depression in a large meta-analysis are the corpus callosum, which connects the two hemispheres, and the corona radiata, a segment of the limbic-thalamo-cortical circuitry involved in emotion regulation (van Velzen *et al*. 2020). However, the authors point towards a structural disconnectivity in depressed individuals as the revealed alterations in WM microstructure in adults with MDD are rather widespread. Alterations in these tracts can be observed through magnetic resonance diffusion tensor imaging (DTI), which presents a non-invasive technique investigating WM architecture *in vivo* based on the tissue water diffusion rate. Most frequently studied is fractional anisotropy (FA), which is regarded as a quantitative index of WM coherence. FA quantifies directional diffusion from zero ( = isotropic) to one ( = anisotropic/constrained along one axis). High FA values are interpreted as revealing a highly organised and normally myelinated axon structure. In contrast, decreased FA might represent reduced coherence in the main preferred diffusion direction and accordingly reflect WM dysfunction.

To the best of our knowledge, only few DTI studies, with small sample sizes, examined participants with familial risk. On the one hand, there is evidence of decreased FA in at-risk participants, pointing towards family history as a risk factor (Bracht, Linden, & Keedwell, 2015; Huang, Fan, Williamson, & Rao, 2011; Keedwell *et al*. 2012), implying that healthy at-risk groups resemble diagnosed patients on a neurobiological level. On the other hand, Frodl *et al*. (2012) provided evidence for greater FA in healthy controls (HC) at-risk as compared to low-risk HC. This study did not include MDD patients, but the results suggest a compensation mechanism which might be further used to distinguish healthy at-risk participants from already-affected at-risk MDD patients.

In sum, there are contradictory reports on WM alterations associated with familial risk, and a lack of DTI studies which analyse (1) healthy as well as depressed individuals (2) with and without familial risk for MDD, (3) including adult, not only adolescent participants, (4) while controlling for the closely related, and therefore potentially confounding, factor environmental risk, as previous studies have found distinct WM correlates of childhood maltreatment (Frodl *et al*. 2012; Huang, Gundapuneedi, & Rao, 2012; Meinert *et al*. 2019). Therefore, the objective of the present study was to investigate the familial risk for MDD and its associations with WM microstructure in a large well-characterised sample of HC and MDD with additional consideration of childhood maltreatment. Based on multiple previous findings (Huang *et al*. 2012; Meinert *et al*. 2019; Repple *et al*. 2020; van Velzen *et al*. 2020), we expect a main effect of diagnosis, more specifically, reduced FA in MDD compared with HC, specifically in the left SLF. We further expect that healthy relatives of MDD patients might show adaptation on a neural level, i.e., increased FA, despite the familial risk as they have already entered adulthood and passed the critical age range in adolescence according to findings by Weissman *et al*. (2016b). Lastly, we explore the effects of childhood maltreatment as an environmental component of familial risk by correcting our findings for the degree of self-reported maltreatment experiences.

## Materials and methods

### Participants

The present sample was drawn from the bicentric Marburg-Münster Affective Cohort Study (MACS/FOR2107-cohort) which has been previously described in more detail elsewhere (Kircher *et al*. 2019; see Vogelbacher *et al*. 2018, for the MRI quality assurance protocol). It consisted of *N* = 528 participants (*N* = 401 female, *M_age_* = 31.26, s.d.*_age_* = 11.72), aged from 18 to 65 years with West-European ancestry; *N* = 262 HC compared with *N* = 266 patients with a lifetime diagnosis of MDD. Furthermore, we divided these two groups into two similarly-sized risk and low-risk groups, respectively: *N* = 129 HC (HCr) and *N* = 132 MDD with familial risk for MDD (MDDlr), and *N* = 133 HC (HClr) and *N* = 134 MDD without (MDDlr) (see online Supplement S1 for further information on sample selection). We operationalised familial risk as reporting at least one first-degree relative (biological mother, father, siblings and/or children) with a known history of past or current psychological treatment due to a diagnosis of MDD, while no other primary psychiatric diagnoses were allowed in these relatives. In contrast, we only included participants in the low-risk groups (HClr and MDDlr), if, reportedly, no first-degree relative had ever been diagnosed with a mental disorder.

Recruitment was implemented through psychiatric hospitals, newspapers and flyers. All groups were matched for age, site (Marburg/Münster), sex and years of education using ‘MatchIt’ in R^®^ (2020, Version 4.0.1) and hence did not differ significantly with respect to these variables (Table 1). Participants were excluded if there was any history of neurological (e.g. concussion, stroke, tumour, neuro-inflammatory diseases) or medical (e.g. cancer, chronic inflammatory or heart diseases) conditions, or substance dependence.

**Table 1.** Sociodemographic characteristics of the total sample (*N* = 528)

Characteristic	HC (*n* = 262)		MDD (*n* = 266)		Test statistics			
	HClr (*n* = 133)	HCr (*n* = 129)	MDDlr (*n* = 134)	MDDr (*n* = 132)	Across all groups	HC v. MDD	HCr v. HClr	MDDr v. MDDlr
Sex, m/f	35/98	33/96	29/105	30/102	χ^2^(3) = 1.09^a^	–	–	–
Site: Marburg/Münster	64/69	64/65	53/81	67/65	χ^2^(3) = 4.15^a^	–	–	–
Age	31.03 ± 11.74	31.78 ± 12.51	29.97 ± 10.83	32.27 ± 11.83	*F*_(3,524)_ = 0.97^b^	–	–	–
Education, in years	14.05 ± 2.40	13.84 ± 2.21	13.62 ± 2.39	13.67 ± 2.63	*F*_(3,524)_ = 0.85^b^	–	–	–
CTQ	31.32 ± 8.54	32.22 ± 7.90	41.77 ± 16.09	44.67 ± 14.05	–	*t*(526) = −10.84***^c^	*t*(260) = −0.89^d^	*t*(264) = −1.34^d^
BDI^e^	2.61 ± 2.18	3.00 ± 2.35	17.67 ± 10.94	16.56 ± 11.56	–	*t*(525) = −20.31***^c^	*t*(260) = −1.40^d^	*t*(263) = 0.80^d^
SIGH-ADS^f^	1.6 ± 2.36	2.1 ± 2.38	12.43 ± 8.74	11.59 ± 9.46	–	*t*(512) = −17.27***^c^	*t*(257) = −1.70^d^	*t*(253) = 0.74^d^

Abbreviations: HC, healthy controls; MDD, major depressive disorder; HClr, low-risk HC; HCr, at-risk HC; MDDlr, low-risk MDD; MDDr, at-risk MDD; CTQ, Childhood Trauma Questionnaire; BDI, Beck Depression Inventory; SIGH-ADS, Structured Interview Guide for the Hamilton Depression Rating Scale with Atypical Depression online Supplement.

a χ^2^-test.

b One-way analysis of variance (ANOVA) assuming equal variance.

c *t* test for independent samples, assuming unequal variance.

d *t* test for independent samples, assuming equal variance.

e Data not available for one MDD participant.

f Data not available for 14 participants.

*Note.* Numbers represent respective *n*, or Mean ± Standard Deviation.

*** *p* < 0.001, two-tailed.

The Structured Clinical Interview (SCID; Wittchen, Wunderlich, Grushwitz, & Zaudig, 1997) was employed by trained personnel to assess whether participants fulfilled standardised criteria defined by the Diagnostic and Statistical Manual of Mental Disorders IV (DSM-IV-TR; American Psychiatric Association, 2000) for a lifetime diagnosis of MDD. The Beck Depression Inventory (BDI; Beck, Ward, Mendelson, Mock, & Erbaugh, 1961) was administered additionally to assess the presence and severity of current (subclinical) depressive symptomatology, while all HCs with BDI score ⩾ 10, considering the cut-off for none or minimal depression, were excluded (Beck, Steer, & Carbin, 1988). HCr v. HClr as well as MDDr v. MDDlr did not differ significantly in BDI scores (Table 1).

For an overview of antidepressant medication intake, we calculated a Medication Load Index (MedIndex) for every MDD participant, i.e., the sum of absent medication ( = 0), equal or lower than the average dose ( = 1), or higher than the average dose ( = 2) for each psychopharmacological agent. This is an established method (Hassel *et al*. 2008; Redlich *et al*. 2014) considering the active ingredient and the daily dose intake recommended by the Physician's Desk Reference (Reynolds, 2008). Since age of onset of MDD (van Velzen *et al*. 2020) and lifetime cumulative duration of depressive episodes (De Diego-Adeliño *et al*. 2014) have been shown to be significantly associated with disruption of WM, it is important to note that the two MDD groups did not differ significantly with respect to these variables. Furthermore, MedIndex, remission status, and recurrency of episodes were evenly distributed across the two groups (Table 2).

**Table 2.** Clinical characteristics and medication in the MDD sample (*n* = 266)

Characteristic	MDDr (*n* = 132)	MDDlr (*n* = 134)	Test statistic	*p*
Clinical				
Lifetime psychiatric comorbidity	56	64	χ^2^(1) = .765	0.382^a^
Recurrent episodes	87	73	χ^2^(1) = 3.63	0.057^a^
Remission status: Acute/partially/fully remitted	50/31/51	70/25/39	χ^2^(2) = 5.56	0.062^a^
Lifetime cumulative duration of depressive episodes [months]^b^	41.67 ± 72.62	29.32 ± 42.01	*t*(227) = 2.85	0.144^c^
Number of lifetime depressive episodes^d^	3.46 ± 6.75	2.59 ± 2.70	*t*(253) = 2.47	189^c^
Duration of current episode [months]^e^	17.54 ± 26.34	13.34 ± 19.52	*t*(156) = 1.72	0.252^c^
Age of onset^f^	21.6 ± 9.52	22.78 ± 9.89	t(263) = 1.00	0.320^a^
Medication				
Agomelatine	6	9	χ^2^(1) = 0.59	0.443^a^
Tricyclic Antidepressant	9	5	χ^2^(1) = 1.27	0.260^a^
Noradrenaline dopamine reuptake inhibitor	5	4	χ^2^(1) = 0.13	0.717^a^
Selective Serotonin Reuptake Inhibitor	33	32	χ^2^(1) = 0.05	0.832^a^
Serotonin-Norepinephrine Reuptake Inhibitor	33	30	χ^2^(1) = 0.25	0.616^a^
Noradrenergic and specific serotonergic antidepressant	9	7	χ^2^(1) = 0.30	0.585^a^
Hypericum perforatum	1	2	χ^2^(1) = 0.32	0.570^a^
Benzodiazepines	0	2	χ^2^(1) = 1.99	0.159^a^
No antidepressant medication	52	60	χ^2^(1) = 0.79	0.374^a^
MedIndex	1.16 ± 1.18	1.22 ± 1.41	*t*(264) = 0.41	0.685^g^

Abbreviations: MDD, major depressive disorder; MDDlr, low-risk MDD; MDDr, at-risk MDD; MedIndex, Medication Load Index.

a χ^2^-test (two-tailed).

b Data available for *n* = 229.

c *t* test for independent samples (two-tailed), assuming equal variance.

d Data available for *n* = 255.

e Only available for patients in their acute stage, *n* = 158.

f Data missing for one participant ^g^ *t* test for independent samples (two-tailed), assuming unequal variance.

*Note.* Numbers represent respective *n*, or Mean ± Standard Deviation.

Concerning childhood maltreatment, HCr v. HClr as well as MDDr v. MDDlr, respectively, did not differ significantly (all *ps* > 0.05) in the total score of the German version of the Childhood Trauma Questionnaire (CTQ; Wingenfeld *et al*. 2010) (Table 1).

The study protocol for this cohort was approved by the ethics committee of the Medical Faculties, University of Marburg (AZ: 07/14) and University of Münster (AZ: 2014-422-b-S). The procedure was performed in accordance with the ethical guidelines and regulations. All participants provided written informed consent and received financial compensation for participation.

### DTI data acquisition

All participants were examined on a 3 T whole body MRI scanner (Marburg: Tim Trio, Siemens, Erlangen, Germany; Münster: Prisma fit, Siemens, Erlangen, Germany) with a GRAPPA acceleration factor of 2 and identical sequence parameters for both sites: Fifty-six axial slices, 2.5-mm thick with no gap, were measured with cubic voxels of 2.5 mm edge length (TE = 90 ms, TR = 7300 ms). Five non-diffusion-weighted (DW) images (*b_0_* = 0) and 2 × 30 DW images with a b-value of 1000 s/mm^2^ for spatial directions were acquired. These scan parameters were consistent over the two sites. GRE field mapping: 2:01; DTI 1 & 2 each 4:10; DTI b0 3x 0:31; reverse phase encoding 0:53; in total, the DTI acquisition lasted 10 min and 46 s.

### Image processing

Preprocessing and analyses were conducted with FSL6.0.1 [http://fsl.fmrib.ox.ac.uk/fsl/fslwiki/, FMRIB, Oxford Center for Functional MRI of the Brain, University of Oxford, Department of Clinical Neurology, John Radcliffe Hospital, Oxford, United Kingdom (Jenkinson, Beckmann, Behrens, Woolrich, & Smith, 2012; Smith *et al*. 2004; Woolrich *et al*. 2009)]. DTI image processing methods and quality controls were already described in Meinert *et al*. (Meinert *et al*. 2019). Briefly, the DW images were corrected for motion artefacts and eddy currents with FSL's ‘eddy’ (Andersson & Sotiropoulos, 2016), with a subsequent rotation of *b*-vectors. As reference for alignment, the first *b_0_* was used after automated skull stripping. For this purpose, FMRIB's brain extraction tool (Smith, 2002) was applied prior to fitting a diffusion tensor model at each voxel using ‘DTIFIT’ within FMRIB's Diffusion Toolbox (FDT) (Behrens *et al*. 2003). For quality assurance of our data, the open-source software DTIPrep (Oguz *et al*. 2014) was used with default options with an automatic pipeline. Gradients failing checks for intensity-related artefacts are deleted. Intensity artefacts are gradients with a large deviation from the mean of all gradients and were subsequently deleted. As a result, affected images up until a threshold of ⩽ 20% were deleted, in which case the participant was excluded from further analyses. This resulted in a number of 64.32 images on average for our sample [s.d. = 1.31, range: (54–65)].

FA is the most frequently reported measure of diffusion in DTI studies besides mean diffusivity (MD), radial diffusivity (RD), and axial diffusivity (AD). We focus on FA with the intention of making our results comparable to previous studies in this area. Our analyses on MD, RD, and AD are available in more detail in the online Supplements. These additional measures contribute to findings of FA providing information about tissue microstructure alterations. MD is a parameter of overall diffusion, averaged across the three eigenvalues of the diffusion tensor, frequently denoted as the apparent diffusion coefficient. RD reflects diffusion radial to the axons, whereas AD is associated with diffusion parallel to the axons (Alexander, Lee, Lazar, & Field, 2007; Feldman, Yeatman, Lee, Barde, & Gaman-Bean, 2010).

### Analyses

Demographic and clinical data were analysed with the Statistical Package for Social sciences (IBM SPSS Statistic 27; SPSS Inc., Chicago, IL, USA). Tract-based spatial statistics (TBSS) (Smith *et al*. 2006) were applied for the reduction of partial volume effects and registration misalignments. We corrected for multiple comparisons with Threshold-Free Cluster Enhancement (TFCE) with 5000 permutations. Additionally, estimated cluster sizes were corrected for the family-wise error (FWE) at *p* < 0.05. Since data were acquired with two different MRI scanners, and due to a body coil exchange in Marburg, two dummy-coded variables (Marburg pre body-coil: yes/no, Marburg post body-coil: yes/no) with Münster as reference category were created (Vogelbacher *et al*. 2018).

In a first step, analyses of covariance (ANCOVAs) were conducted in FSL with FA (as well as AD, RD and MD, respectively) as dependent variables, and diagnosis as well as state of familial risk as independent variables to estimate the main effect of diagnosis and familial risk as well as their interaction. Nuisance variables were age, sex, total intracranial volume (TIV), Marburg pre body-coil, and Marburg post body-coil. We investigated the main effect of diagnosis, and the main effect of risk as well as the interaction effect of diagnosis × risk (*F* tests). In case of significant results, post-hoc pairwise *t* tests were calculated to investigate individual group differences and the direction of the effects. For analyses in FSL, effect sizes were calculated based on the *t* value of the peak voxel provided by FSL and respective sample sizes according to Cooper, Hedges, & Valentine (2009).

Furthermore, we conducted accessory analyses and robustness checks: In two sub-analyses, we excluded (a) participants under the age of 26, aiming to confirm results in a sample which has presumably passed the critical age range for developing MDD according to Weissman *et al*. (2016b), and (b) participants of or above the age of 60 to rule out effects of age on FA (Salat *et al*. 2005; Sexton *et al*. 2014; Walhovd *et al*. 2005; Westlye *et al*. 2010). Next, we conducted control analyses for potential effects of outliers using SPSS by extracting individual mean FA values of the significant cluster from the main analysis. In the total sample, we examined outliers with Cook's distance > 3 s.d. and recalculated the identical general linear model (GLM) in SPSS. Furthermore, we investigated the influence of childhood maltreatment including CTQ_Sum_ as a covariate. For analyses in SPSS, given effect sizes in *η*^2^_p_ were converted into Cohen's d for comparability. In addition, we conducted an accessory sensitivity analysis in which we only included participants in the risk groups whose parents had been treated for MDD (online Supplement S5).

## Results

### Main analysis: ANCOVA with main effect of diagnosis and risk, and their interaction

**Fig. 1.**
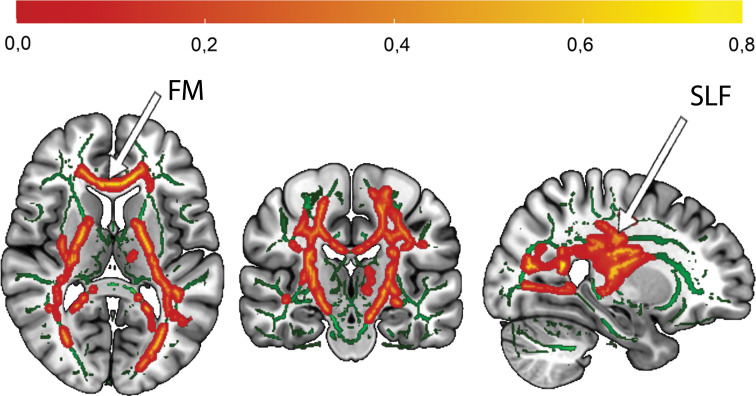
Affected white matter tracts by effect of diagnosis (HC > MDD). *Note.* Increased FA in healthy controls as compared to patients with major depressive disorder, mainly in the forceps minor and superior longitudinal fasciculus, *p*_tfce_-_FWE_ = 0.009. In order to illustrate the effects on the FMRIB58 template (visualised in green) for all three sectional views in the Montreal Neurological Institute (MNI) Atlas coordinate system, the mean FA value was obtained from FA values of all significant voxels (*p*_tfce−FWE_ < 0.05). Red-yellow areas represent voxels in significant clusters, using the ‘fill’ command in FSL. The colour bar indicates the probability of a voxel being a member of the different labelled regions within the JHU-atlas, averaged over all the voxels in the significant mask (*p*_tfce−FWE_ < 0.05). MNI coordinates of selected plane: *x* = 26, *y* = −19, *z* = 10. FM, forceps minor; SLF, superior longitudinal fasciculus

**Figure 2.**
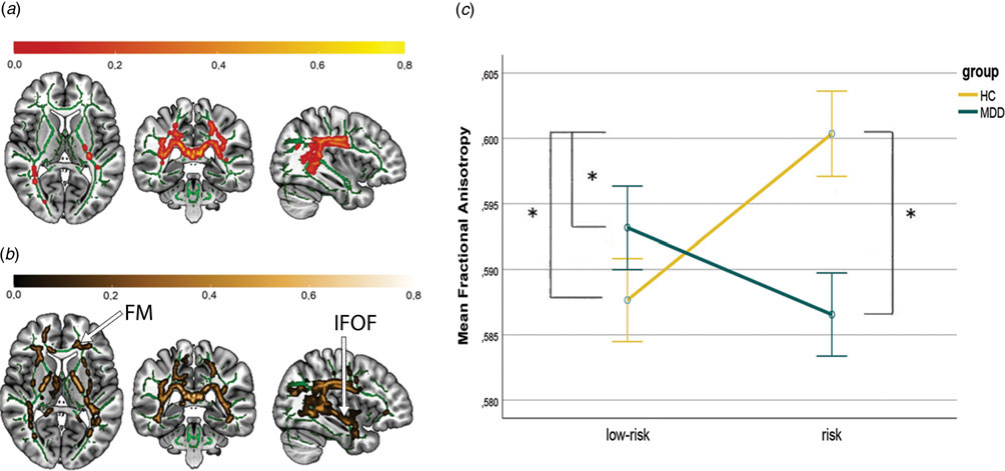
Interaction effect of diagnosis and familial risk, and effect of familial risk in HC. *Note.* Affected white matter tracts by A: Interaction effect of diagnosis × familial risk, *p_tfce−_*_FWE_ = 0.036. B: Familial risk in HC. Widespread increased FA in HCr as compared to HClr, *p_tfce−_*_FWE_ < 0.001. Effects illustrated on the FMRIB58 template (visualised in green) in the Montreal Neurological Institute (MNI) Atlas coordinate system. Red-yellow areas represent voxels in significant clusters, using the ‘fill’ command in FSL. The colour bar indicates the probability of a voxel being a member of the different labelled regions within the JHU-atlas, averaged over all the voxels in the significant mask (*p*_tfce−FWE_ < 0.05). MNI coordinates of selected plane for A and B: *x* = 37, *y* = −37, *z* = 7.C: Significant increase in FA in HCr as compared to all other groups. Error bars represent standard errors of the mean. Asterisk represents statistical significance (*p_tfce−FWE_* < 0.05) in post-hoc-*t*-tests. The mean FA value was obtained from FA values of all significant voxels (*p*_tfce−FWE_ < 0.05). FM, Forceps Minor; IFOF, inferior fronto-occipital fasciculus.

A significant main effect of diagnosis was found (*p*_tfce_-_FWE_ = 0.021, total *k* = 1774 voxels in 3 clusters, peak voxel of a largest cluster: *x* = 27, *y* = −23, *z* = 16). A post-hoc *t* test revealed that individuals from both HC groups had higher FA compared with both MDD groups in the forceps minor (FM) and the left SLF with a moderate effect size (*d* = 0.40, *p*_tfce_-_FWE_ = 0.009, total *k* = 19 792 voxels in 4 clusters, peak voxel of largest cluster: *x* = 24, *y* = −20, *z* = 6, Fig. 1). Significant effects were also found on RD (*p*_tfce_-_FWE_ = 0.033) but not on MD (*p*_tfce_-_FWE_ = 0.314), with increased RD in MDD patients (online Supplement S2). The main effect of familial risk was not significant neither for FA (*p*_tfce*−*FWE_ > 0.265), nor RD (*p*_tfce_-_FWE_ > 0.129), nor MD (*p*_tfce_-_FWE_ > 0.125). However, the interaction of diagnosis × familial risk yielded significant results (*p_tfce−_*_FWE_ = 0.036, total *k* = 9297 voxels in 5 clusters, peak voxel of largest cluster: *x* = 26, *y* = −21, *z* = 28). Clusters predominantly in the left corticospinal tract (CST) and the bilateral SLF were affected by altered FA (Fig. 2a). The effect was also significant for MD (*p*_tfce_-_FWE_ = 0.042) and RD (*p*_tfce_-_FWE_ = 0.008) (online Supplement S2). No significant main or interaction effects were found for AD (all *p*_tfce_-_FWE_ > 0.115).

Post-hoc pairwise *t* tests revealed a significant difference between the two HC groups, with widespread increased FA values in the HCr group (*p_tfce−_*_FWE_ < 0.001, total *k* = 41 494 voxels in one cluster, peak voxel: *x* = 37, *y* = −37, *z* = 12), mainly in the FM and right inferior fronto-occipital fasciculus (IFOF), with a moderate effect size (*d* = 0.59) (Fig. 2b). Furthermore, HCr also had significantly higher FA values compared to the MDDr and MDDlr group (MDDr: *p_tfce−_*_FWE_ < 0.001, total *k* = 42 419 voxels in one cluster, peak voxel: *x* = 28, *y* = −18, *z* = 21, *d* = 0.67; MDDlr: *p_tfce−_*_FWE_ < 0.005, total *k* = 34 734 voxels in one cluster, peak voxel: *x* = −15, *y* = −9, *z* = −6, *d* = 0.61). For location and size of all significant clusters as well as affected tracts see online Supplementary Tables S2 and S3. All other effects were not significant (all *p*_tfce−FWE_ > 0.332; online Supplementary Table S3).

### Accessory analyses and robustness checks

Analysing only participants with ⩾ 26 years of age (*N* = 284) the post-hoc differences between healthy risk and low-risk participants found in the original sample could be replicated with a nominally higher effect size (*p*_tfce−FWE_ = 0.009, *d* = 0.75) (online Supplement S3). Similarly, including participants with < 60 years of age yielded the same results (online Supplement S4). The results from our first analyses remained also unchanged when excluding participants whose children or siblings were affected (online Supplement S5).

Control analyses in SPSS confirmed our main results. Even after excluding 14 outliers from the original sample, the main effect of diagnosis and interaction effect remained significant (online Supplement S4). A weak but significantly negative correlation between CTQ_Sum_ and FA scores (*r*(526) = −0.094, *p* = 0.031) did not abolish the effect either: when including CTQ_Sum_ as a covariate in the general linear model in SPSS, the main effect (*F*_(1,527)_ = 4.28, *p* = 0.039, *d* = 0.18) and interaction effect (*F*_(1,527)_ = 36.18, *p* < 0.001, *d* = 0.53) were still significant with no significant impact of CTQ (*F*_(1,527)_ = 0.325, *p* = 0.569).

## Discussion

In the present study, we investigated cross-sectional correlates of familial risk for MDD and WM microstructure in a large sample of HC and patients with MDD. We were (a) able to replicate a previously reported significant main effect of diagnosis and (b) found a significant interaction effect of familial risk with diagnosis. HC had increased FA as compared to MDD participants in the FM and left SLF. The interaction effect was driven by widespread increased FA in healthy participants with a first-degree relative with MDD as compared to a low-risk healthy sample, mainly in the FM and right IFOF. The additional covariance of self-reported retrospective childhood maltreatment scores did not change the significant results, suggesting the reported WM alterations reflect rather genetic than environmental risk which has also been associated with distinct disruptions in WM microstructure (Frodl *et al*. 2012; Huang *et al*. 2012; Meinert *et al*. 2019), effects on cognition (Goltermann *et al*. 2021) and grey matter anomalies (Opel *et al*. 2016). No significant differences were found between MDDr and MDDlr concerning all four analysed diffusion indices while recurrence of episodes and state of remission did not differ between the MDD samples.

We were able to replicate results by Murphy & Frodl (2011) indicating decreased FA in MDD participants across different states of remission in the left SLF as compared to HC. Connecting the frontal cortex with the parietal, temporal and occipital cortex, the SLF is a major bidirectional association tract which is involved in specific behavioural and cognitive functions in healthy adults such as verbal memory, but also non-verbal cognition, working memory, and visuo-spatial functions (Koshiyama *et al*. 2020), as well as recognition of emotional faces (Ioannucci, George, Friedrich, Cerliani, & Thiebaut de Schotten, 2020). A meta-analysis across emotional disorders identified reduction in FA in the SLF, as compared to HC, as the most replicable finding and concludes that the functions associated with the SLF are in line with impaired perception of and attention to emotional information in these disorders (Jenkins *et al*. 2016). Consolidation of WM fibre microstructure in the SLF might be positively associated with emotional perception and cognitive control in HC as compared to MDD in general.

In contrast to previous studies, no significant reduction in FA in our healthy risk v. low-risk participants were found in the bilateral cingulum bundles (Bracht *et al*. 2015; Huang *et al*. 2011; Keedwell *et al*. 2012). Instead, the present increases in FA in HCr as compared to HClr are partly in contrast to previous studies with a similar design, revealing decreases in FA. Nevertheless, our results are in line with Frodl *et al*. (2012) who also reported increased FA in the right IFOF, among other tracts, in healthy relatives of MDD patients as compared to HC without familial risk. These structural differences could result in differences in the neural processing of emotion. The IFOF is involved in emotional visual function, building an association between the vision-related ventro-medial occipital to the emotion-related infero- and dorsolateral regions of the frontal lobe (Catani, Howard, Pajevic, & Jones, 2002). Altered emotional visual perception, i.e., altered transmission of valenced signals linked to reduced FA in IFOF has been observed in depression (Kieseppä *et al*. 2010) and female adolescent HCr (Joormann, Cooney, Henry, & Gotlib, 2012). We also found increased FA in the FM in HCr v. HClr. The FM connects the two hemispheres, more specifically the dorsolateral prefrontal cortices (DLPFC). The DLPFC is involved in attention, executive functions and internally guided behaviour during goal-oriented and working memory tasks (Kane & Engle, 2002). Furthermore, it is activated during emotion regulation processes (Dixon, Thiruchselvam, Todd, & Christoff, 2017; Versace *et al*. 2018), more specifically mindfulness-based negative emotion regulation strategies (Opialla *et al*. 2015). Enhanced WM fibres in the IFOF and FM might point towards enhanced negative emotion regulation and activation of cognitive strategies in HCr which prevent depressive symptomatology and promote mental health.

A reason why we found results contradicting previous studies might be a different age range of participants. Importantly, Huang *et al*. (2011) included adolescents from age 12 to 20 only, whereas our sample consisted of individuals between 18 and 65, with the majority of participants in an age range from 25 to 30. Weissman *et al*. (2016b) found that the risk for first onset of major depression in high-risk and low-risk individuals was highest between ages 15 and 25. In our reported sub-analysis excluding participants from our sample aged under 26 years, we were indeed able to replicate the findings of increased FA in HCr v. HClr with a higher effect size than in the original analysis. Our participants might therefore have adapted to the increased risk: Increased FA in HCr might reflect overcompensation, a neurological mechanism allocating more, yet protective, resources in tracts connecting areas which are involved in emotion-processing, emotion regulation and executional tasks. Extending the results of Frodl *et al*. (2012), we revealed an interaction between diagnosis and familial risk, suggesting that our HCr group is resilient in contrast to MDDr. However, this should be addressed in future studies with measurements of resilience, e.g., questionnaires.

### Limitations

Our study's cross-sectional design does not allow disentangling between precursor and consequence. Longitudinal studies are needed to account for putative causal relationships between familial risk factors and alterations in WM microstructure.

Even though reported in the follow-up study by Weissman *et al*. (2016a, 2016b), studies have exposed contradictory findings on the age of onset for MDD (Kessler *et al*. 2005; Solmi *et al*. 2021). However, we based our decision to conduct an accessory analysis with an older age group with this specific cut-off on data in high-risk and low-risk individuals which were investigated over a time period of 30 years.

Secondly, we relied on participants' self-report on whether a first-degree relative suffered from MDD. In order to increase the level of confidence regarding diagnosis, we only included participants who confirmed that the affected relative received treatment related to the diagnosis, but however, we cannot exclude that participants categorised as low-risk might have been unaware of affected relatives. Including only participants whose relatives were treated for depression might also indicate more severe cases of depression, and might also be influenced by the social and financial ability to seek and receive treatment. Moreover, we did not control for the number of affected relatives. Relatedly, family history and personal depression are not independent. On the one hand, the experience of having a first-degree relative with MDD might lead to more attention in the individual towards this disorder. The individual potentially reports symptoms earlier and seeks treatment faster, as they are aware of the increased risk. On the other hand, self-experienced stigma and shame might lead to not mentioning affected family members.

Furthermore, our operationalisation of familial risk does not only represent an estimation of genetic, but also of a stressful familial environment. Future studies should investigate this association between genetic markers and WM microstructure. However, childhood maltreatment scores as measured with the CTQ_Sum_ did not differ significantly between HClr and HCr. Additionally, the interaction effect remained stable when including CTQ_Sum_ as a covariate in our analyses, making strong influences of the environmental component of familial risk rather unlikely. However, disentangling the specific mechanisms of neglect and abuse as mediators on developing depression needs to be addressed in future studies with specific study designs including groups recruited for presence/absence of childhood maltreatment.

Notably, there were more females than males in our sample. Further studies on the interaction of sex and familial risk on FA would be of interest.

## Conclusion

The main finding of our study shows that resilient individuals with familial risk for MDD exhibit widespread increased FA, with an emphasis on the forceps minor and right IFOF as compared to healthy individuals without such risk. This challenges previous results that HC at risk have decreased FA in the cingulum bundle. The effects remained significant after the exclusion of participants under the age of 26, and when correcting for a measure of environmental risk, i.e., self-reported childhood maltreatment. Even though we were able to replicate results indicating increased FA in HC in the left SLF as compared to MDD participants, the significant interaction of familial risk and diagnosis suggests adaptation processes on a neural level in a healthy, but not in an already affected at-risk sample, possibly reflecting resilience. Future longitudinal studies are needed to disentangle precursor and consequence of these WM alterations in resilient individuals and to which degree they can help understand the biological basis of depression, particularly taking the rather modest effect sizes into account.

## Supplementary material

For supplementary material accompanying this paper visit https://doi.org/10.1017/S003329172200188X. click here to view supplementary material
