# Supplementary material for: Familial risk for major depression: differential white matter alterations in healthy and depressed participants
Source: Psychol Med. 2022 Sep 2;53(11):4933–42. doi: 10.1017/S003329172200188X (PMC10476061; doi:10.1017/S003329172200188X)
Supplement: Supplementary file 1 [file S003329172200188Xsup001.docx]

# **Supplementary Information**

## Supplement 1: Choosing participants from the FOR2107 cohort

Data without dropouts
*N*=1741
924 HC, 817 MDD

HC with secured familial risk for MDD and DTI data surviving quality checks

*N*=134

Total Sample

*N*=528

*N*=129 HCr, *N*=133 HClr, *N*=132 MDDr, *N*=134 MDDlr

MDD with and without familial risk, HC without risk matched in age, gender, education and site to this sample

HC with BDI≥10 excluded

*N*=6

Post-hoc dropout of *N*=2 MDDr (data freeze update)

3^rd^ datafreeze (March 11th, 2021)

*N*=2478

Dropout of missing data or diagnoses irrelevant for the current analysis (*N*=442)

Total data
*N*=2037
1050 HC, 987 MDD

Clinical dropout criteria (e.g., severe somatic illness, MRI contraindication, substance abuse/dependence) (*N*=296)

The third data freeze (March 11^th^, 2021) from the FOR2107 cohort was used comprising *N*=2478 participants, of which *N*=986 patients suffered from MDD and *N*=1050 were HC. Participants with diagnoses irrelevant for the present study (e.g., Bipolar Disorder), or missing data, were excluded. In a next step, dropout criteria such as severe somatic illness, MRI contraindications or substance dependence/abuse were applied which led to a basic sample of *N*=1741 with *N*=924 HC and *N*=817 MDD. As our reference sample, we first chose participants with a secured familial risk of MDD, while no other diagnoses in first-degree relatives were allowed, leading to a sample of *N*=134 from which DTI data was available and survived quality checks. Then, we matched the other three groups for age, site, sex and education stepwise to this group. Five participants from the HCr group had a BDI score≥9 and were therefore excluded afterwards. Due to a datafreeze update, two individuals from the MDDr dropped out post-hoc. The final sample consisted of *N*_HClr=_133, *N*_HCr_=129, *N*_MDDlr_=134, *N*_MDDr_=132.

To optimize the harmonization of recruitment across the two sites, various measurements were taken, e.g., uniform standard operational procedures (SOP), video ratings/trainings for inter-rater reliability, quality checks of clinical data and a MRI quality assurance protocol. Interview ratings were implemented by trained PhD students who underwent standardized training, while interviews were regularly supervised and videotaped. These videos were rated by all other raters on a regular basis. The overall intra-class correlations (ICC) across all videotaped interviews were HAMD (ICC = 0.918), HAMA (ICC = 0.959), YMRS (ICC = 0.829), SANS (ICC = 0.845), and SAPS (ICC = 0.923). Potential mismatches of ratings were discussed and fine-tuned in weekly conferences. Furthermore, regular site visits took place to promote the exchange of procedures and to keep the process constant. This is described in more detail in Kircher et al. (2019). For the comprehensive quality assurance (QA) for assessing the general quality of the MRI data, to detect potential malfunctions in the scanning equipment, and to evaluate inter-site differences that need to be accounted for in subsequent analyses, a protocol was established (Vogelbacher et al., 2018).

## Supplement 2: Effects in RD and MD

We found a significant effect on RD for main effect of diagnosis, with increased RD values in MDD as compared to HC (p_tfce-FWE_=.033, total *k*=4866 voxels in 4 clusters, peak voxel of largest cluster: *x*=19, *y*=-7, *z*=7). Clusters in the bilateral corticospinal tracts (CST) were predominantly affected. The interaction effect yielded significant results for MD (*p*_tfce-FWE_=.042) and RD (*p*_tfce-FWE_=.009). This was explained by increased MD contrasting HClr, MDDlr and MDDr vs. HCr, respectively. The effects were consistently visible in the bilateral SLF. Similar results were revealed for RD, with increased values in HClr, MDDr and MDDlr as compared to HCr. Clusters mainly in the bilateral SLF, the FM and the left IFOF yielded significant results. For location of peak voxels, number of clusters and *p*-values see Supplementary Table 1, for affected tracts see Supplementary Table 2.

## Supplement 3: Excluding participants aged 25 and below

From the original sample, we excluded participants with an age of 25 years and below, leading to a sub-sample of HClr *n*=69, (M_age_=38.90, SD_age_=11.53), HCr *n*=66 (M_age_=40.61, SD_age_=11.95), MDDlr *n*=68, M_age_=37.31, SD=10.76), MDDr *n*=81 (M_age_=38.48, SD_age_=11.18). The four groups did not differ significantly with regard to age (*F*(3,280)=0.972, *p*=.407). State of remission was not significantly differently distributed across MDDr and MDDlr (ꭓ²(2)=3.537, *p*=.171). The same ANCOVA as in analysis 1 was conducted in FSL with FA as dependent variable, and diagnosis as well as state of familial risk as independent variables to estimate the main effect of diagnosis and familial risk as well as their interaction. Nuisance variables were age, sex, TIV, Marburg pre body-coil, and Marburg post body-coil. We were able to partly replicate the results from analysis 1: The main effect of diagnosis was not significant anymore (*p*_tfce-FWE_ =.318), but the interaction effect (*p*_tfce-FWE_<.01, total *k*=9072 voxels in 3 clusters, peak voxel of largest cluster: *x*=17, *y*=-44, *z*=22). The main effect of risk remained non-significant (*p*_tfce-FWE_=.609). Post hoc *t*-tests revealed that HCr exhibited higher FA than HClr, predominantly in the right IFOF and bilateral SLF (*p*_tfce-FWE_=.009, total *k*=23759 voxels in 3 clusters, peak voxel of largest cluster: *x*=-17, *y*=-6, *z*=5, *d*=0.75, based on peak voxel). HCr had significantly higher, widespread, FA than MDDr (*p*_tfce-FWE_=.002, total *k*=30234 voxels in 1 cluster, peak voxel: *x*=30, *y*=-42, *z*=17, *d*=0.81, based on peak voxel), but not as compared to MDDlr, even though there was a trend towards statistical significance (*p*_tfce-FWE_=.065). All other post hoc *t*-tests did not reach level of significance (all *ps*_tfce-FWE_>.111). The interaction effect was stable even when including childhood maltreatment represented by CTQ_Sum_ as a covariate on top of age, sex, TIV, bodycoil pre/post in SPSS in a general linear model (GLM) using group (HC vs. MDD) and familial risk (low-risk vs. risk) and their interaction as fixed factors, mean FA extracted from the interaction effect from FSL as dependent variable (*F*(1,283)=23.07, *p*<.001, *d*=0.58). In this model, CTQ_Sum_ did not have a significant impact as a covariate (*F*(1,283)=0.65, *p*=.352).

## Supplement 4: Excluding participants aged 60 and above

Excluding participants of 60 years or older, a sample size of *N*=515; *N*_HClr_=131, *N*_HCr_=123, *N*_MDDlr_=133, *N*_MDDr_=128 remained. There were still no differences neither in age (*F*(3,511)=.499, *p*=.683) nor in education years (*F*(3,511)=1.043., *p*=.373), nor in site (ꭓ²(1)=3.957, *p*=.266) nor sex (ꭓ²(1)=1.252, *p*=.741). Across the two healthy groups, no differences in CTQ_Sum_ (*t*(252)=-1.593, two-sided *p*=.113, assuming equal variance), BDI (*t*(252)=-.908, two-sided *p*=.365, assuming equal variance) arose as a consequence to this reduction of participants. In the MDD samples, CTQ_Sum_ did not differ significantly (*t*(258)=.604, two-sided *p*=.546, assuming equal variance), BDI (*t*(259)=-1.277, two-sided *p*=.203, assuming equal variance). State of remission was not significantly differently distributed across MDDr and MDDlr (ꭓ²(2)=.4.870, *p*=.088)

In FSL, the identical model as in analysis 1 was recalculated with FA as dependent variable, familial risk and group as independent variables as well as age, sex, TIV, and BCpre, BCpost as covariates. The main effect of group was significant (*p*_tfce-FWE_=.013) with significant clusters in the right anterior thalamic radiation, right corticospinal tract, and forceps minor. The interaction effect remained significant (*p*_tfce-FWE_=.005), with significant clusters in the left corticospinal tract, and bilateral SLF. As before, the main effect of risk was not significant (*p*_tfce-FWE_=.226). The post-hoc *t*-tests showed the same pattern as in our main analysis, with the HCr group differing significantly from the three other groups: HCr>HClr (*p*_tfce-FWE_=.001) with significant clusters mainly in the forceps minor and right IFOF; HCr>MDDr (*p*_tfce-FWE_<.001) showing widespread alterations, mainly in the forceps minor; HCr>MDDlr (*p*_tfce-FWE_=.001), with significant clusters in the forceps minor, bilateral IFOF; MDDr>HCr (*p*_tfce-FWE_=.990), MDDlr>MDDr (*p*_tfce-FWE_=.278), MDDr>MDDlr (*p*_tfce-FWE_=.810), MDDlr>HCr (p_tfce-FWE_=.917), MDDr>HClr (*p*_tfce-FWE_=.613), HClr>MDDr (*p*_tfce-FWE_=.364), HClr>MDDlr (*p*_tfce-FWE_=.461), MDDlr>HClr (*p*_tfce-FWE_=.491), HClr>HCr (*p*_tfce-FWE_=.965).

## Supplement 5: Including at-risk participants with depressed parents only

Excluding participants whose affected relatives were children or siblings led to a remaining sample size of *N*=451, with *N*_HClr_=133, *N*_HCr_=96, *N*_MDDlr_=134, *N*_MDDr_=88. There were still no differences neither in age (*F*(3,447)=.464, *p*=.707) nor in education (*F*(3,447)=.1.357, *p*=.255), nor in site (ꭓ²(1)=3.427, *p*=.330) nor sex (ꭓ²(1) =2.357, *p*=.502). Across the two healthy groups, no differences in CTQ_Sum_ (*t*(227)=-.448, two-sided *p*=.655, assuming equal variance) or BDI (*t*(227)=-1.325, two-sided *p*=.186, assuming equal variance) arose as a consequence to this reduction of participants. The same results applied to the two MDD samples CTQ_Sum_ (*Z*=-.442, *p*=.659), BDI (*t*(219)=.285, two-sided *p*=.386, assuming equal variance). State of remission was not significantly differently distributed across MDDr and MDDlr (ꭓ²(2)=2.388, *p*=.303).

In FSL, the identical model as in analysis 1 was recalculated with FA as dependent variable, familial risk and group as independent variables as well as age, sex, TIV, and BCpre, BCpost as covariates. Due to the reduction of sample size, the (undirected) main effect of diagnosis was not significant (*p*_tfce-FWE_=.071), while, however, the directed *t*-test HC>MDD was (*p*_tfce-FWE_=.024), following our initial hypothesis. Voxels in the right corticospinal tract, the forceps minor and the right SLF were mainly affected. The main effect of risk was not significant (*p*_tfce-FWE_=.118). The interaction effect remained significant (*p*_tfce-FWE_=.006), with widespread clusters, prominently in the bilateral corticospinal tract, the right IFOF, the right inferior longitudinal fasciculus, and the right SLF. The results of the post-hoc tests followed the same pattern as in analysis 1, HCr>MDDr (*p*_tfce-FWE_<.001) with voxels mainly affected in the forceps minor, and bilateral SLF; HCr>HClr (*p*_tfce-FWE_=.002) with significant clusters in the right IFOF; HClr>HCr (*p*_tfce-FWE_=.886); MDDlr>MDDr (*p*_tfce-FWE_=.362); MDDr>MDDlr (*p*_tfce-FWE_=.776), MDDlr>HCr (*p*_tfce-FWE_=.766); HCr>MDDlr (*p*_tfce-FWE_=.007) with significant clusters in the right corticospinal tract, forceps minor, left IFOF, and bilateral SLF; MDDr>HCr (*p*_tfce-FWE_=.819); MDDr>HClr (*p*_tfce-FWE_=.669); HClr>MDDr (*p*_tfce-FWE_=.451); HClr>MDDlr (*p*_tfce-FWE_=.532); MDDlr>HClr (*p*_tfce-FWE_=.386).

## Supplement 6: Outlier analysis using Cook’s distance for FA

Cook’s distance was calculated in SPSS for a GLM using group (HC vs. MDD) and familial risk (low-risk vs. risk) and their interaction as fixed factors, mean FA extracted from the interaction effect (*F*-test) as dependent variable, as well as age, sex, TIV, bodycoil pre/post as covariates (*M_Cook_*=.002, *SD_Cook_*=.004, Range: ]0, .027]). Outliers were defined as extreme values of the Cook’s distance (*M_Cook_*+3**SD_Cook_*). This resulted in the exclusion of 14 participants (5 HClr, 3 HCr, 5 MDDlr, 1 MDDr). The same GLM was calculated repeatedly in SPSS, without these participants. The main effect of diagnosis (*F*(1,513)=10.77, *p*=.001, *d*= 0.29) as well as the interaction effect (*F*(1,513)=41.54, *p*<.001, *d*=0.57) remained significant. The main effect of risk reached level of significance as well (*F*(1,513)=4.75, *p*=.030, *d*=0.19).

## Supplementary Table 1

*Location and size of all significant clusters from Analysis 1 (*N*=528)*

| \| Cluster / contrast \| *p*_tfce-FWE_ \| k \| x \| y \| z \| \| --- \| --- \| --- \| --- \| --- \| --- \| \| Fractional anisotropy \|  \|  \|  \|  \|  \| \| HCr > HClr   1. Cluster \| .001 \| 41494 \| 37 \| -37 \| 12 \| \| HCr > MDDlr   1. Cluster \| .005 \| 34734 \| -15 \| -9 \| -6 \| \| HCr > MDDr   1. Cluster \| <.001 \| 42419 \| 28 \| -18 \| 21 \| \| Main effect of diagnosis  HC vs. MDD  HC > MDD \| .021  .009 \| 1774  19628 \| 27  26 \| -23  -19 \| 16  10 \| \| Interaction effect  Diagnosis × familial risk  1. Cluster  2. Cluster  3. Cluster  4. Cluster  5. Cluster \| .049  .048  .048  .042  .004 \| 14  44  52  585  8602 \| 28  42  41  34  26 \| -36  -23  -21  -62  -21 \| 40  -18  -7  25  28 \| \| Radial diffusivity \|  \|  \|  \|  \|  \| \| HClr > HCr   1. Cluster \| .005 \| 46722 \| 28 \| -15 \| 27 \| \| MDDr > HCr   1. Cluster \| .001 \| 33959 \| 27 \| -27 \| 20 \| \| MDDlr > HCr   1. Cluster 2. Cluster 3. Cluster 4. Cluster 5. Cluster 6. Cluster 7. Cluster 8. Cluster 9. Cluster 10. Cluster 11. Cluster \| .05  .05  .05  .05  .05  .05  .05  .05  .044  .009  .009 \| 3  4  5  11  12  13  16  34  324  13200  15805 \| -15  -50  -44  -54  -44  -6  -37  -7  -22  20  -15 \| -40  -19  27  -20  21  14  -3  18  -52  -28  -9 \| 7  -18  11  -17  17  -13  36  -12  12  37  -6 \| \| Main effect of diagnosis  MDD > HC   1. Cluster 2. Cluster 3. Cluster 4. Cluster \| .05  .043  .037  .03 \| 11  184  2180  2491 \| -31  -24  -15  19 \| -36  -77  -8  -7 \| 39  12  -4  7 \| \| Interaction effect  Diagnosis × familial risk   1. Cluster \| .008 \| 8986 \| 25 \| -21 \| 30 \| \| Mean Diffusivity \|  \|  \|  \|  \|  \| \| HClr > HCr   1. Cluster 2. Cluster 3. Cluster 4. Cluster 5. Cluster 6. Cluster 7. Cluster 8. Cluster 9. Cluster 10. Cluster 11. Cluster 12. Cluster 13. Cluster 14. Cluster 15. Cluster 16. Cluster 17. Cluster 18. Cluster 19. Cluster 20. Cluster 21. Cluster 22. Cluster 23. Cluster 24. Cluster 25. Cluster 26. Cluster 27. Cluster 28. Cluster 29. Cluster \| .05  .05  .05  .05  .05  .05  .05  .05  .05  .05  .05  .05  .05  .05  .05  .05  .05  .05  .05  .05  .05  .05  .05  .05  .049  .05  .049  .049  .008 \| 1  1  1  1  1  2  2  2  2  5  5  5  6  6  9  11  13  20  23  27  30  36  46  46  55  70  73  107  37652 \| -11  -30  11  -50  -31  39  30  -14  -24  -47  -32  -13  -8  41  39  -34  -6  2  12  -25  -16  33  -10  43  31  60  -52  -10  31 \| 27  -29  -74  -6  4  -78  -21  -60  -19  -7  -76  -50  22  -55  -55  -49  -26  28  21  -9  -71  -84  -51  -48  10  -41  -45  -81  -43 \| -6  1  6  -25  18  18  -3  52  62  -25  6  49  -7  38  3  13  24  0  20  31  13  10  52  36  -8  -1  20  24  16 \| \| MDDr > HCr   1. Cluster \| .011 \| 18374 \| 20 \| -24 \| 37 \| \| MDDlr > HCr   1. Cluster 2. Cluster 3. Cluster 4. Cluster 5. Cluster 6. Cluster 7. Cluster 8. Cluster \| .05  .05  .05  .05  .049  .041  .033  .034 \| 1  4  5  5  56  805  3773  6022 \| -22  44  -19  -22  23  31  42  -20 \| -57  12  -65  -59  -22  -14  -10  -9 \| 31  12  35  32  -3  12  27  8 \| \| Interaction effect  Diagnosis × familial risk   1. Cluster 2. Cluster 3. Cluster 4. Cluster \| .05  .05  .047  .042 \| 3  15  87  282 \| 15  30  20  17 \| -34  -44  -40  -18 \| 28  19  29  34 \|   *Abbreviations:* HC=healthy controls; MDD=patients with major depressive disorder; HCr=at-risk HC, HClr=low-risk HC; MDDr=at-risk MDD; MDDlr=low-risk MDD; k=voxel count, x-y-z=location in 3D Montreal Neurological Institute (MNI) space. |
| --- | --- | --- | --- | --- | --- | --- | --- | --- | --- | --- | --- | --- | --- | --- | --- | --- | --- | --- | --- | --- | --- | --- | --- | --- | --- | --- | --- | --- | --- | --- | --- | --- | --- | --- | --- | --- | --- | --- | --- | --- | --- | --- | --- | --- | --- | --- | --- | --- | --- | --- | --- | --- | --- | --- | --- | --- | --- | --- | --- | --- | --- | --- | --- | --- | --- | --- | --- | --- | --- | --- | --- | --- | --- | --- | --- | --- | --- | --- | --- | --- | --- | --- | --- | --- | --- | --- | --- | --- | --- | --- | --- | --- | --- | --- | --- | --- | --- | --- | --- | --- | --- | --- | --- | --- | --- | --- | --- | --- |

## Supplementary Table 2

*Probability that affected voxels belong to tracts assigned by the JHU white-matter tractography atlas*

| Region | FA | RD | MD |  |
| --- | --- | --- | --- | --- |
| HC vs. MDD  (main effect diagnosis) | HC>MDD | HC<MDD | HC<MDD |  |
| ATR L  ATR R  **Corticospinal tract L**  **Corticospinal tract R**  Cing. (cingulate gyrus) L  Cing. (cingulate gyrus) R  Cing. (hippocampus) L  Cing. (hippocampus) R  Forceps major  **Forceps minor**  IFOF L  IFOF R  ILF L  ILF R  **SLF L**  SLF R  Uncinate fasciculus L  Uncinate fasciculus R  SLF (temporal part) L  SLF (temporal part) R | 1.4549  0.8433  1.9635  1.9162  0.3078  0.0124  0.0112  0.0277  1.7267  **4.7371**  1.7491  1.5587  1.1158  1.0906  **2.7146**  1.3367  0.4228  0.0681  1.2417  0.4770 | 1.3159  1.5911  **4.5756**  **6.1318**  --  --  --  --  --  --  0.1783  0.0698  0.1434  0.0039  1.4884  0.4089  --  --  0.2074  0.0930 | --  --  --  --  --  --  --  --  --  --  --  --  --  --  --  --  --  --  --  -- |  |
| Interaction effect  Diagnosis × familial risk | FA | RD | MD |  |
| ATR L  ATR R  **Corticospinal tract L**  **Corticospinal tract R**  Cing. (cingulate gyrus) L  Cing. (cingulate gyrus) R  Cing. (hippocampus) L  Cing. (hippocampus) R  Forceps major  IFOF L  IFOF R  ILF L  ILF R  **SLF L**  **SLF R**  Uncinate fasciculus L  SLF (temporal part) L  SLF (temporal part) R | 0.3268  0.0474  **2.4894**  1.4310  0.0288  0.2532  0.0017  0.0195  0.6469  0.1617  1.2151  0.1279  0.8417  **3.5064**  **4.8129**  0.0474  1.5944  1.8671 | 0.3701  0.1767  **2.4099**  1.7712  0.0504  0.2076  0.0018  0.0124  0.3454  0.6087  0.4284  0.5530  0.2164  **3.9991**  **5.0813**  0.0636  1.8675  1.9814 | **--**  0.1207  --  **10.1552**  **--**  0.0517  --  --  --  --  0.2931  --  0.0690  --  0.0517  --  --  -- |  |
| HCr vs. HClr | FA  (HCr>HClr) | RD  (HCr<HClr) | MD  (HCr<HClr) |  |
| ATR L  ATR R  Corticospinal tract L  Corticospinal tract R  Cing. (cingulate gyrus) L  Cing. (cingulate gyrus) R  Cing. (hippocampus) L  Cing. (hippocampus) R  Forceps major  **Forceps minor**  IFOF L  **IFOF R**  ILF L  ILF R  **SLF L**  **SLF R**  Uncinate fasciculus L  Uncinate fasciculus R  SLF (temporal part) L  SLF (temporal part) R | 1.2854  1.4070  1.2224  1.0710  0.1159  0.2556  0.1144  0.1808  0.9883  **2.2397**  1.7246  **2.0472**  1.3072  1.1061  1.5500  1.9002  0.6291  0.4032  0.7185  0.6907 | 1.0055  1.0893  1.0366  0.9851  0.2428  0.1907  0.0091  0.0076  0.9577  **2.1076**  1.5670  1.9378  1.2821  1.1768  1.9122  1.9967  0.5509  0.3409  0.8730  0.7454 | 0.5852  0.4793  0.5041  0.6375  0.2910  0.0889  0.0032  0.0065  0.8878  **2.5949**  1.4509  1.7069  1.3841  1.1494  **2.2286**  **2.2767**  0.4196  0.2734  0.9798  0.8380 |  |
| HCr vs. MDDlr | FA  (HCr>MDDlr) | RD  (HCr<MDDlr) | MD  (HCr<MDDr) |  |
| ATR L  ATR R  Corticospinal tract L  Corticospinal tract R  Cing. (cingulate gyrus) L  Cing. (cingulate gyrus) R  Cing. (hippocampus) L  Cing. (hippocampus) R  Forceps major  **Forceps minor**  **IFOF L**  IFOF R  ILF L  ILF R  **SLF L**  **SLF R**  Uncinate Fasciculus L  Uncinate Fasciculus R  **SLF (temp. part) L**  SLF (temp. part) R | 1.6737  1.0123  1.2713  1.2941  0.2114  0.0937  0.0718  0.0222  1.1353  **3.3192**  **2.0439**  1.9653  1.3350  0.9895  1.9120  1.9749  0.7148  0.2495  0.8716  0.6081 | 1.1117  0.5976  1.4083  1.4145  0.3060  0.1345  0.0137  0.0105  1.0593  1.7662  **2.3925**  1.4411  1.6899  1.4019  **2.3509**  **2.4341**  0.8260  0.0384  0.9828  0.7409 | 1.4779  0.2652  1.7079  1.4307  --  0.0007  --  0.0007  0.0472  0.0067  **3.0569**  1.7970  **2.4232**  1.8816  **5.0584**  **4.6846**  1.0921  0.0876  **2.1243**  1.3581 |  |
| HCr vs. MDDr | FA  (HCr>MDDr) | RD  (HCr<MDDr) | MD  (HCr<MDDr) |  |
| ATR L  ATR R  Corticospinal tract L  Corticospinal tract R  Cing. (cingulate gyrus) L  Cing. (cingulate gyrus) R  Cing. (hippocampus) L  Cing. (hippocampus) R  Forceps major  **Forceps minor**  IFOF L  **IFOF R**  ILF L  ILF R  **SLF L**  **SLF R**  Uncinate fasciculus L  Uncinate fasciculus R  SLF (temporal part) L  SLF (temporal part) R | 1.3539  1.6025  1.3091  1.2891  0.2758  0.1661  0.0086  0.0114  1.0873  **3.4746**  1.4054  1.6512  1.0659  1.0220  1.9849  1.9518  0.3983  0.2227  0.9149  0.7714 | 0.8738  0.7030  1.4396  1.2806  0.2977  0.2116  0.0049  0.0123  1.3056  **3.2481**  1.5218  1.8093  1.1840  1.1356  **2.4481**  **2.3447**  0.4199  0.1495  1.1197  0.9329 | 0.2058  0.2360  1.5797  1.2185  0.0639  0.0543  0.0009  0.0118  1.4475  --  1.1651  **2.0989**  1.4383  1.4899  **3.3017**  **3.1300**  0.0166  0.0057  1.3345  1.2443 |  |

*Abbreviations:* FA=fractional anisotropy; L=left, R=right; RD=radial diffusivity; MD=mean diffusivity; HC=healthy controls; MDD=patients with major depressive disorder; HCr=at-risk HC, HClr=low-risk HC; MDDr=at-risk MDD; MDDlr=low-risk MDD; ATR=anterior thalamic radiation; Cing.=Cingulum; IFOF=inferior fronto-occipital fasciculus; ILF=inferior longitudinal fasciculus; SLF=superior longitudinal fasciculus; UF=uncinate fasciculus

## Supplementary Table 3

*Non-significant contrasts from Analysis 1 (*N*=528)*

| Contrast from post hoc *t*-test | *p*_tfce-FWE_ | | |
| --- | --- | --- | --- |
|  | FA | RD | MD |
| HClr > HCr | .955 | -- | -- |
| MDDlr > MDDr | .332 | .493 | .292 |
| MDDr > MDDlr | .797 | .351 | .414 |
| MDDlr > HCr | .867 | -- | -- |
| MDDr > HClr | .583 | .509 | .626 |
| HClr > MDDr | .502 | .412 | .24 |
| HClr > MDDlr | .510 | .395 | .333 |
| MDDlr > HClr | .400 | .526 | .365 |
| HCr > HClr | -- | .998 | .972 |
| HCr > MDDr | -- | .986 | .936 |
| HCr > MDDlr | -- | .945 | .988 |
| MDDr > HCr | .984 | -- | -- |
| HC < MDD  At-risk vs. low-risk | --  .265 | --  .129 | .136  .125 |

*Abbreviations*: HC=healthy controls; MDD=patients with major depressive disorder; HCr=at-risk HC, HClr=low-risk HC; MDDr=at-risk MDD; MDDlr=low-risk MDD; FA=fractional anisotropy; RD=radial diffusivity; MD=mean diffusivity

## References

Kircher, T., Wöhr, M., Nenadic, I., Schwarting, R., Schratt, G., Alferink, J., … Dannlowski, U. (2019). Neurobiology of the major psychoses: a translational perspective on brain structure and function—the FOR2107 consortium. *European Archives of Psychiatry and Clinical Neuroscience*, *269*(8), 949–962. https://doi.org/10.1007/s00406-018-0943-x

Vogelbacher, C., Möbius, T. W. D., Sommer, J., Schuster, V., Dannlowski, U., Kircher, T., … Bopp, M. H. A. (2018). The Marburg-Münster Affective Disorders Cohort Study (MACS): A quality assurance protocol for MR neuroimaging data. *NeuroImage*, *172*, 450–460. https://doi.org/10.1016/j.neuroimage.2018.01.079
